# Immediate analgesic effect of acupuncture for acute primary dysmenorrhea: protocol of a randomized controlled trial

**DOI:** 10.3389/fmed.2025.1644666

**Published:** 2025-09-10

**Authors:** Gaoyangzi Huang, Wenjun Li, Qifu Li, Xin Tang, Siwen Zhao, Shumin Zhang, Ruqin Yang, Zili Liu, Taipin Guo, Jun Qian

**Affiliations:** ^1^School of Second Clinical Medicine/The Second Affiliated Hospital, Yunnan University of Chinese Medicine, Kunming, China; ^2^Department of Psychiatry, Anning City First People's Hospital, Affiliated with Kunming University of Science and Technology, Kunming, China

**Keywords:** acute, primary, dysmenorrhea, immediate, analgesic, effect, acupuncture, randomized controlled trial

## Abstract

**Background:**

Primary dysmenorrhea (PD) is a common gynecological disorder that significantly affects women’s work and personal lives, especially in acute episodes when immediate pain relief is often needed. The objective of this study is to evaluate the immediate analgesic effect of acupuncture for acute PD within 10 min.

**Methods:**

This is a randomized sham-controlled clinical trial involving 80 participants with acute PD who will be randomized in a 1:1 ratio into the Acupuncture group and the Sham acupuncture group. Each participant will receive 10 min acupuncture session. For participants in the Sham acupuncture (SA) group, the Park Sham Acupuncture Device (PSD) and blunt needles will be used to simulate treatment without skin penetration. The visual analog scale (VAS) will be evaluated before treatment and at 0–1, 2, 4, 6, 8, and 10 min after treatment. The primary outcome is the effective analgesia rate after 10 min of acupuncture. Secondary outcomes include effective analgesia rate at other time points (0–1, 2, 4, 6, and 8 min), VAS, blinding assessment, and the treatment effectiveness expectations scale. Adverse events during each treatment period will be collected and recorded. All outcomes will be analyzed on an intention-to-treat. The recruitment for this study is expected to be completed between May 1, 2024, and September 1, 2025.

**Discussion:**

The primary need of patients with PD is the immediate relief of pain. This study aims to investigate the immediate analgesic effect of acupuncture on acute PD. The results of this study will provide a rapid and reliable clinical basis for the use of acupuncture in the treatment of PD, potentially offering a non-pharmacological alternative for pain management that could reduce reliance on medications and their associated side effects.

**Clinical trial registration:**

Identifier ChiCTR2300070826. This study has been registered at the Chinese Clinical Trial Registry (https://www.chictr.org.cn).

## Introduction

Primary Dysmenorrhea (PD) is a functional pain disorder without organic pathology, characterized by severe cramping lower abdominal pain during or before menstruation (1). It has been a common gynecological condition that troubles women, and globally, the prevalence among women of reproductive age ranges from 16 to 94% (2). Severe cases may be accompanied by additional symptoms such as nausea, vomiting, diarrhea, weakness, and even fainting (3). Moderate to severe acute PD can be debilitating for women of childbearing age, reducing their quality of life, affecting their productivity at work and school (4), and potentially causing additional psychological burdens (5, 6). In Europe, PD results in the loss of 3.6 million quality-adjusted life years annually, which is comparable to the impact of chronic conditions such as asthma or chronic migraines (7).

Non-steroidal anti-inflammatory drugs (NSAIDs) and hormonal drugs are currently the first choice for the clinical treatment of acute PD (8). However, these drugs are accompanied by some side effects, such as the potential for digestive disorders, headaches, and drowsiness. In general, analgesic drugs begin to take effect after about 15 min (9), and some medications used for treating acute PD may have subtle or delayed effects (10). The primary and urgent need for patients is immediate relief from pain during each PD episode. However, the dependence on medications and their side effects leads to hesitation in medication choices. Therefore, developing rapid, efficient, and low-risk treatment methods is the focus of current research.

With the increasing research on nonpharmacologic therapies, acupuncture has become a widely accepted approach in pain management (11, 12), demonstrating positive and significant effects in alleviating acute pain (13–19). Acupuncture has also shown clinical effectiveness in treating acute PD (18, 19), but there is a lack of rigorously standardized randomized controlled trials (RCTs) to support this. Therefore, we have designed this RCT to investigate the immediate analgesic effects of a 10-min acupuncture intervention in acute PD. The 10-min observation period is selected because prior studies suggest that acupuncture’s peak analgesic effect typically occurs within 5–10 min of treatment initiation (20–22). This duration also balances clinical feasibility and patient compliance, avoiding the fatigue and inattention associated with longer sessions.

## Materials and methods

### Trial strategy

**Figure 1:**
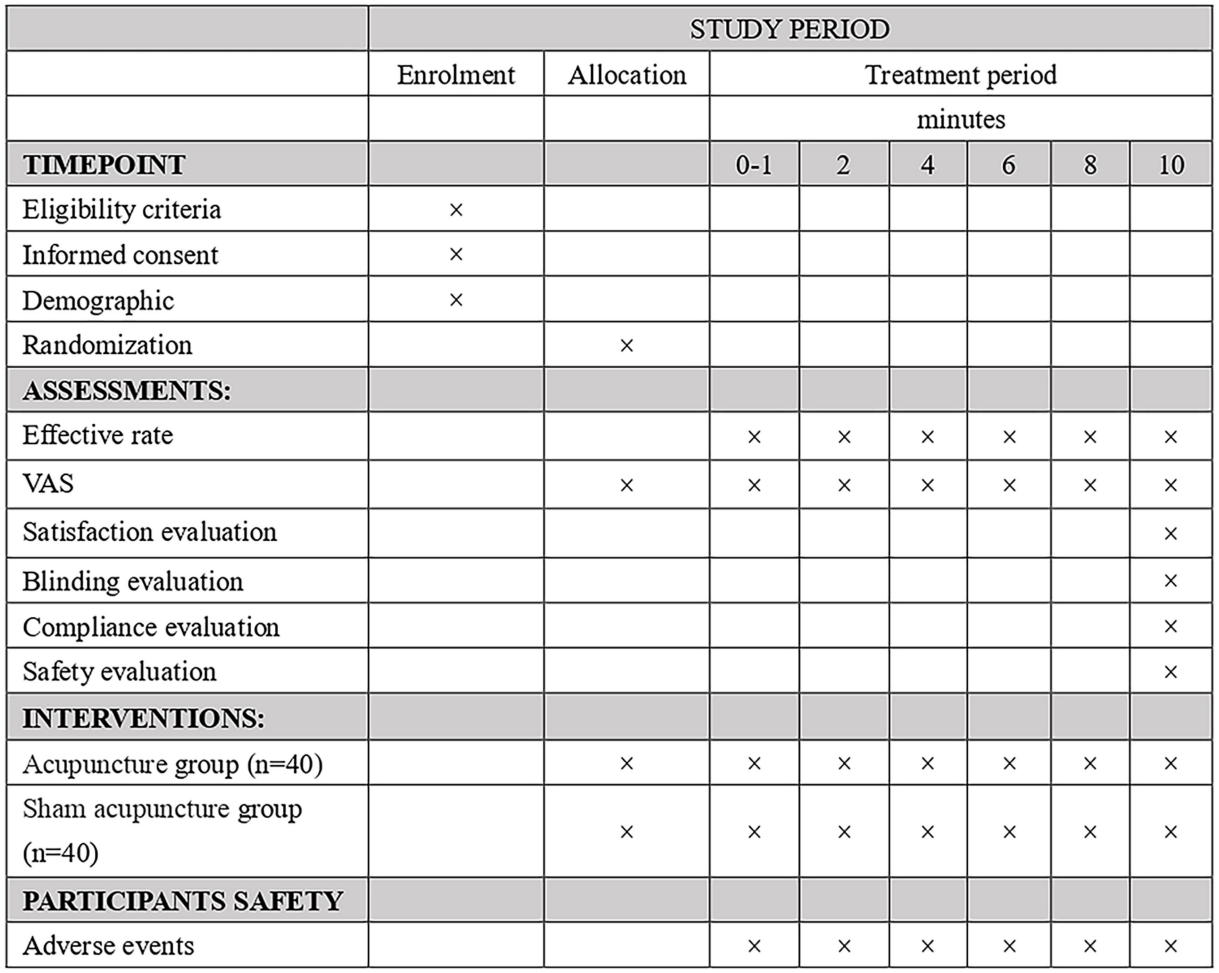
Study schedule for data measurements.

**Figure 2:**
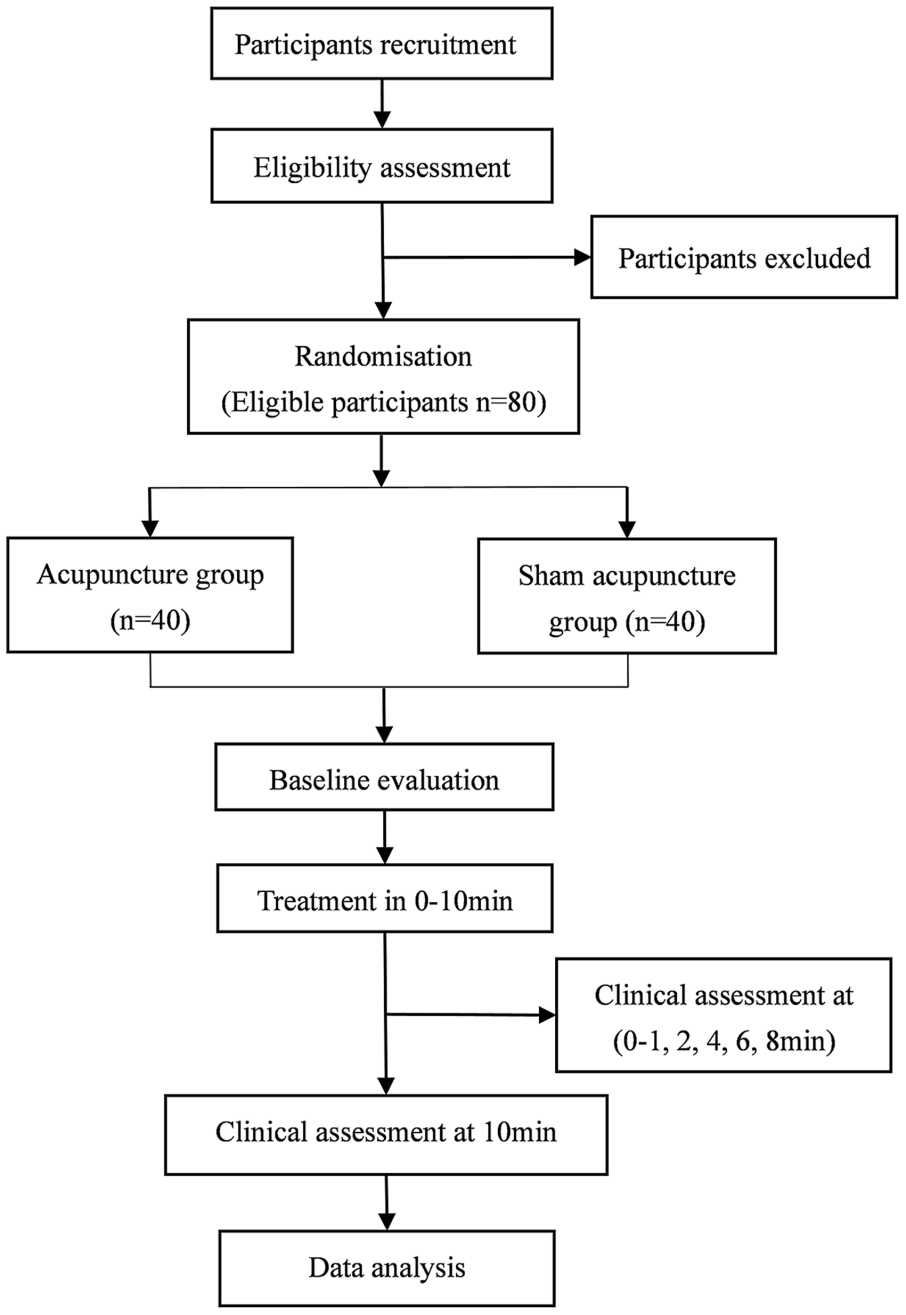
Flowchart of the study design.

The study protocol is designed following the Declaration of Helsinki principles and the Standard Protocol Items: Recommendations for Interventional Trials guidelines (SPIRIT 2013) (23). Figure 1 shows the SPIRIT schedule of enrolment, intervention, and assessments. Figure 2 shows the flow chart of the study design is shown in.

This study has been approved by the Ethics Committee of the Second Affiliated Hospital of Yunnan University of Chinese Medicine (approval number: 2022–010). The study was registered on the Chinese Clinical Trial Registry (ChiCTR2300070826).

### Recruitment and informed consent

This is a randomized controlled trial (RCT) aimed at evaluating the immediate efficacy of acupuncture in the treatment of acute PD. The study will be conducted at the Second Affiliated Hospital of Yunnan University of Traditional Chinese Medicine, with a planned recruitment of 80 participants diagnosed with PD. A public recruitment advertisement for this study will be designed to recruit the participants online or offline channels, such as the websites, and WeChat public account. Upon meeting the inclusion criteria and expressing willingness to take part in this study, participants will be required to provide written informed consent prior to the commencement of the study (Appendix 1). During the baseline period, participants will be asked to provide information regarding menstrual cycle characteristics (e.g., cycle length, regularity, duration), baseline pain threshold, prior analgesic use, and prior acupuncture experience. These variables will be collected for exploratory analysis of potential confounding effects. Participants retain the right to withdraw from the study at any point, and their personal data will be solely utilized for medical research purposes.

### Eligibility criteria

To be enrolled in this study, the following eligibility criteria, assessed at screening, will be met:

### Inclusion criteria

Participants will be included after meeting all of the following:

1. The diagnostic criteria for PD (24) and experiencing an acute episode.
2. Age 14 to 35 years old.
3. 4 ≤ pain visual analog scale (VAS) score ≤9.
4. Have not received acupuncture treatment or taken analgesic drugs after this episode.
5. Voluntary participation in this study and signed informed consent, with informed family consent required for those under 18 years of age.
6. Have not participated in other PD studies.

### Exclusion criteria

Participants will be excluded if they have any of the following:

1. Combined severe life-threatening primary diseases and other acute abdominal pain.
2. With severe anxiety, depression, insomnia, and other psychiatric disorders.
3. Are afraid of acupuncture, do not cooperate with treatment, or are prone to acupuncture dizziness.

### Withdrawal criteria

1. Cases that do not meet the inclusion criteria or are mistakenly included should be excluded.
2. Patients whose studies are terminated due to severe adverse events or complications, making further treatment inappropriate.
3. Choose to withdraw from their participation in the study.

### Randomization and blinding

Participants will be randomly allocated to either the Acupuncture group or the Sham acupuncture (SA) group in a 1:1 ratio. Random numbers will be generated using SPSS 28.0 (IBM, Chicago, IL, USA, authorization Code: f56b44b8d8e3562ad8a2) by an independent statistician. Randomization information will be placed in opaque sealed envelopes and kept by personnel not involved in the study. Participants will be randomly assigned to different groups and treated in separate rooms to avoid communication. At the end of treatment, the success of the blinding will be assessed using a blinding questionnaire. Two experienced and licensed acupuncturists will participate in the treatment. The two acupuncturists will participate in either the needling group or the sham needling group. This design minimizes the risk of bias from treatment assignment information. To minimize potential bias, the acupuncturists will have no knowledge of the study results and will not be involved in outcome assessment or data analysis. Participants, outcome assessors, and statistical analysts will be unaware of group allocation.

### Interventions

**Figure 3:**
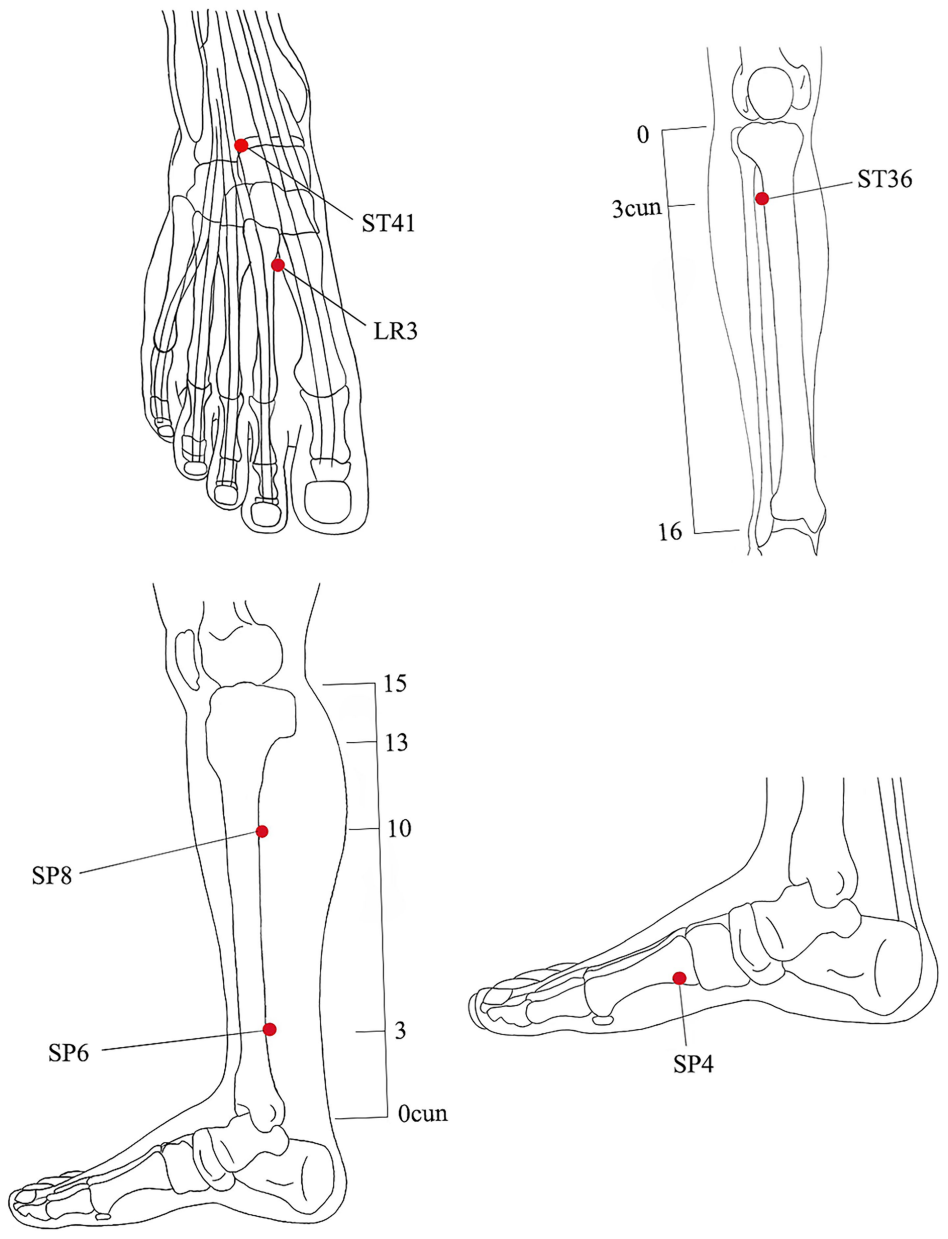
Location of acupoints.

The intervention measures will be in accordance with the Consolidated Standards of Reporting Studies (25) and the Standards for Reporting Interventions in Clinical Studies of Acupuncture (26). Based on the previous literature and clinical experience (18, 27), we selected the dominant acupoints for the treatment of PD as the selected acupoints for this study, including SP4 (Gongsun), ST36 (Zusanli), LR3 (Taichong), SP6 (Sanyinjiao), ST41 (Jiexi), and SP8 (Diji), bilateral. All acupoints will be positioned based on the WHO Standard Acupuncture Point Locations 2010 (ISBN:1101119787117123327). The acupoints are located as shown in Figure 3 and Table 1.

**Table 1:** Location of acupoints.

Acupoints	Location
SP4 (Gongsun)	On the medial aspect of the foot, anteroinferior to the base of the first metatarsal bone, at the border between the red and white flesh.
SP6 (Sanyinjiao)	On the tibial aspect of the leg, posterior to the medial border of the tibia, 3 B-cun superior to the prominence of the medial malleolus.
SP8 (Diji)	On the tibial aspect of the leg, posterior to the medial border of the tibia, 3 B-cun inferior to SP9.
ST36 (Zusanli)	On the anterior aspect of the leg, on the line connecting ST35 with ST41, 3 B-cun inferior to ST35.
LR3 (Taichong)	On the dorsum of the foot, between the first and second metatarsal bones, in the depression distal to the junction of the bases of the two bones, over the dorsalis pedis artery.
ST4 (Jiexi)	On the anterior aspect of the ankle, in the depression at the center of the front surface of the ankle joint, between the tendons of extensor hallucis longus and extensor digitorum longus.

### Appliance selection

**Figure 4:**
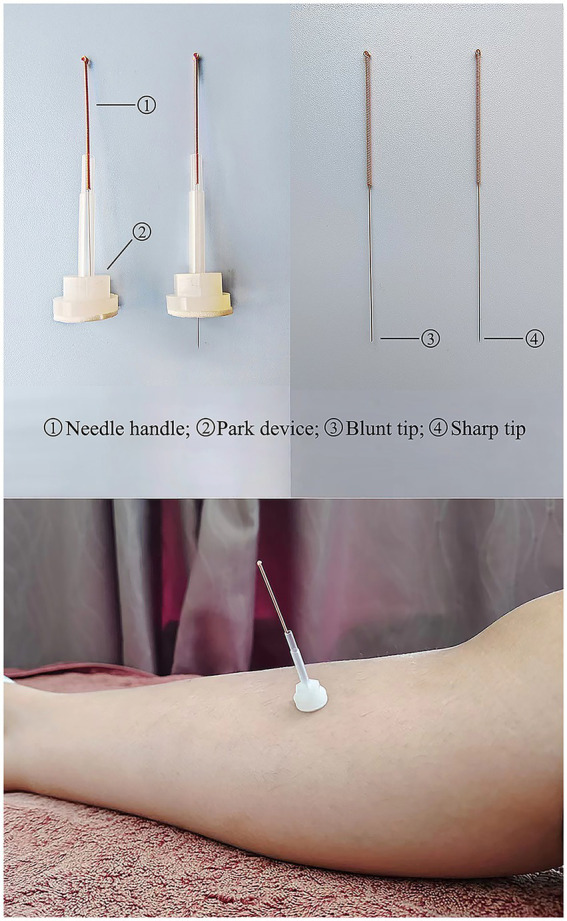
Park sham acupuncture device.

1. Acupuncture needle (Figure 4): use the Huatuo brand disposable acupuncture needles manufactured by Suzhou Medical Supplies Factory, China. Manufacturer’s license number: Su Food and Drug Administration (SFDA) of Machinery Production 20,010,020; Registration certificate number: 201622770970. The acupuncture needle specifications are 0.25 mm × 40 mm.
2. Park Sham Acupuncture Device (PSD) (Figure 4): includes transparent guide tubes (Φ4 × 20 mm, Φ3 × 35 mm), double-sided tape (Φ1 × 15 mm), and opaque plastic bases (Φ4 × 15 mm, Φ5 × 10 mm) sourced from Suzhou Medical Supplies Factory, China (lot number 210401).
3. Blunt needle (Figure 4): use retractable stainless steel blunt needles (0.25 × 40 mm), sourced from Suzhou Medical Supplies Factory, China (lot number 210401).

### Operation

Prior to the procedure, participants will be guided to lie in a supine position, ensuring full exposure of the acupoints. Subsequently, a licensed acupuncturist with a minimum of 3 years of experience will conduct the routine disinfection process. The procedure will then commence.

### Acupuncture group

For participants in the Acupuncture group, PSD and disposable acupuncture needles will be used. After removing the sticker on the base, disposable acupuncture needles are introduced to expose the tips, and the skin is disinfected, the PSD is attached to the acupuncture points, and the acupuncture needles are stuck into the skin for treatment, and all acupoints are gently and straightly pierced by 20 to 30 mm. Lifting and twisting technique, will be employed to elicit the “Deqi” sensation. During the treatment session, a proficient research assistant will promptly gather VAS scores from the subjects at 0–1, 2, 4, 6, 8, and 10 min following the initiation of treatment. The data collection table is shown in Table 2.

**Table 2:** The data collection table.

Use a 10 cm VAS scale with a moving scale between 0 and 10 on the front and a number from 0 to 10 on the back, with 0 being no pain and 10 being the most painful. Please state your pain level according to the following scale. 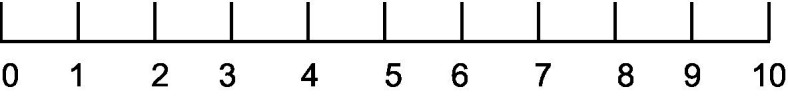 VAS scale (0–10 points) 0: no pain; 1–3: light pain; 4–6: moderate pain; 7–10: severe and unbearable pain.

Projects	Before treatment	Treatment				
	0 min	2 min	4 min	6 min	8 min	10 min
Pain VAS score						

### Sham acupuncture group

For participants in the SA group, PSD and blunt needle will be used. During the procedure, the acupuncturist will have blunt needles touching the skin without penetrating the surface, the participants will feel a slight tingling sensation. The rest of the operation is the same as the Acupuncture group.

### Emergency treatment

During the study, if a participant’s pain persists or escalates to an intolerable level, the trial will be immediately halted, and intervention will be administered promptly. Participants are allowed to take emergency analgesics, such as NSAIDs, under the guidance of a physician, with dosage adjustments made as necessary. Additionally, if participants in the SA group do not experience relief, they will be offered acupuncture treatment or other emergency interventions to ensure the safe and effective delivery of treatment. The treatment details will be meticulously documented on the Case Report Forms (CRFs) by the researchers.

### Primary outcome

The primary outcome is the efficacy rate at 10 min post-treatment. The efficacy rate is calculated as the proportion of participants whose VAS pain scores have decreased by at least 50% compared to the baseline measurement (28).

### Secondary outcomes

The secondary outcomes include the effective rate at various time points (0, 0–1, 2, 4, 6, 8 min), VAS pain score, blinding assessment (assessed by asking participants whether they received true acupuncture and their confidence in this response), and treatment effectiveness expectations scale.

1. The VAS is one of the most commonly used pain intensity assessment tools (Table 2) (29). The scale consists of a 10 cm horizontal line, with one end labeled as ‘0’ to indicate ‘no pain at all’ and the other end labeled as ‘10’ to represent ‘the worst imaginable pain.’ The line presented to participants only displays the two anchor points (‘0’ and ‘10’) without any intermediate numerical markings. Participants will be instructed to mark a position on the line using a pen (e.g., with a dot or an ‘x’) that best represents their current pain intensity at that moment. The VAS will be assessed seven times in total, including at baseline, 0–1 min (during treatment), 2 min (during treatment), 4 min (during treatment), 6 min (during treatment), 8 min (during treatment), and 10 min (at the end of treatment).

After the participants make their mark, the scale will be collected by trained study personnel, who will measure the distance (in millimeters) from the ‘0’ anchor to the marked point using a ruler. This process allows for precise scoring, including fractional scores (e.g., 5.5), based on the exact position of the mark.

Currently, there are no studies specifically targeting the immediate analgesic response in primary dysmenorrhea to determine the minimum clinically important difference (MCID) threshold. Therefore, we combined previous clinical experience and previous studies in acute pain patients, where a decrease of approximately 10–13 mm on the visual analog scale (VAS) can be considered the minimum clinically important difference (MCID) (30).

To ensure uniformity, participants will receive standardized verbal instructions, emphasizing that the marking should reflect their perception of pain without additional guidance or interference. The measurement will be conducted in a quiet and private setting to minimize distractions and ensure accuracy. Study personnel will systematically record the scores to maintain consistency and data integrity.

1. To assess participant blinding effects, participants from the Acupuncture group or SA group will be asked at the end of the treatment whether they believe they received genuine acupuncture. Additionally, they will be required to rate their confidence in the answer on a scale from 0 to 10, where 0 indicates very uncertain and 10 indicates completely certain. If the proportion of correctly identified participants does not significantly exceed 50%, the blind is considered successful (Table 3).
2. The treatment effectiveness expectations scale assesses participants’ expectations regarding the outcomes of acupuncture treatment. Post-treatment, participants will indicate their anticipated treatment outcomes (‘effective’ or ‘ineffective’) and rate, on a scale from 0 to 10, their feelings about receiving positive treatment and the expected results (Table 3).

**Table 3:** Blinding questionnaire and treatment expectations scale.

Blinding test	
Do you think you were given acupuncture treatment or placebo acupuncture?	Yes⬜ No⬜
How sure are you on your answer on a scale of 0 to 10? (0 = very uncertain and 10 = completely certain)	___________________

Treatment expectations scale	
What do you think of the results of your treatment?	Efficiently⬜ Inefficiently⬜
How sure are you on your answer on a scale of 0 to 10? (0 = very uncertain and 10 = completely certain)	___________________

### Sample size

Based on previous study (31) and our past clinical treatment experience, we anticipate that after 10 min of acupuncture treatment, the mean change in VAS score in the acupuncture group will be 4.21 (SD = 1.0), whereas the mean change in VAS score in the sham acupuncture group will be 2.15 (SD = 1.0). With *α* = 0.025 (unilateral, significance level), *β* = 0.1 (type II error rate, or power = 90%), *Δ* = 1.3 (minimum clinically important difference, this is equivalent to a difference of 13 mm on the visual analog scale, which represents the minimum clinically significant change in acute pain.), and k =1 (allocation ratio) (30). The sample size was calculated based on the following formula:


$$n_{C}=\frac{{\left(Z_{1-\alpha }+Z_{1-\beta }\right)}^{2}\sigma^{2}{\left(1+\frac{1}{k}\right)}^{2}}{{\left(\mu_{T}-\mu_{C}-\Delta \right)}^{2}}$$

$\mu_{T}$ : trial group mean; $\mu_{C}:$ control group mean; σ : standard deviation (assuming equal standard deviations for both groups).

Based on the calculations, each group will require at least 36 participants (32). Taking into account an 8% dropout rate, the adjusted sample size will be 40 participants per group. Therefore, we plan to recruit at least 80 participants for this study.

### Statistical analysis

The statistical analysis will be conducted using SPSS 28.0 software. Qualitative data will be represented in percentages or proportions, and quantitative data with a normal distribution will be presented as the mean ± standard deviation, while non-normally distributed data will be expressed as the median and interquartile range. When analyzing variables measured at multiple time points, a repeated measures analysis of variance (ANOVA) will be used. To correct for multiple comparisons, Bonferroni correction will be applied. Effect sizes and 95% confidence intervals (CI) will also be calculated. Analysis of other variables will include independent t-tests or Wilcoxon rank-sum tests, chi-square test or Fisher’s exact tests. The Intention-to-Treat (ITT) principle will be applied to assess baseline characteristics and primary and secondary outcomes. For participants who drop out of the study, missing data will be addressed using multiple imputations by chained equations (MICE), provided the missingness is determined to be missing at random (MAR). This approach aims to ensure the robustness of the analysis and minimize potential biases introduced by missing data. All adverse events (AEs) will be compared between groups using the chi-square test or Fisher’s exact test. A two-sided test will be utilized, with a significance level *p*-value set at 0.05.

### Patient and public involvement

In this study protocol, participants and the general public will not be involved in all stages of the study. The role of participants will be confined to those aspects directly relevant to the core objectives of the research, and they will not be engaged in the recruitment, implementation, or reporting phases of the study. The research findings will be disseminated to participants and the public through educational lectures, brochures, or pamphlets. Moreover, the results will be published in open-access, peer-reviewed journals.

### Data management and confidentiality

Data will be collected by a dedicated investigator who is a member of the research team, and data quality will be monitored by two independent investigators who are not part of the research team. Communication between investigators and subjects will be enhanced to improve adherence and subject retention. Outcome assessors will complete the initial data in the CRFs, which include observation time points, interventions, outcome indicators, and adverse events. Investigators will be asked to follow the requirements of the CRFs and complete the information in a timely and accurate manner. The Ethics Committee of The Second Affiliated Hospital of Yunnan University of Chinese Medicine will consistently review the study’s progress and oversee the collection, allocation, and confidentiality of data. The committee holds the authority to make modifications or terminate the study if necessary. Additionally, an independent data monitoring committee, free from any conflicts of interest with the sponsors, will be responsible for monitoring the study’s data.

### Adverse events reporting and safety monitoring

Throughout the entire research process, participants undergoing acupuncture treatment may experience AEs such as increased pain instead of relief, subcutaneous congestion or hematoma, or even fainting or other severe incidents. Whether these adverse events are directly related to the treatment in this study or not, we will promptly and appropriately address them. In the event of an AE, the participant will receive immediate clinical assessment and necessary medical care. For mild AEs (e.g., minor bruising or needling discomfort), local symptomatic treatment will be provided. For moderate or severe AEs (e.g., vasovagal syncope, severe pain), the intervention will be paused or terminated immediately, and the participant will be closely monitored by qualified medical staff until full recovery. Emergency procedures will be in place to ensure patient safety at all times. Detailed records of all AEs will be documented in CRFs, and a comprehensive report will be submitted to the Ethics Committee of The Second Affiliated Hospital of Yunnan University of Chinese Medicine.

### Ethics and dissemination

This study has received approval from the Ethics Committee of the Second Affiliated Hospital of Yunnan University of Chinese Medicine (Approval Number 2022–010), conducted in accordance with the Helsinki Declaration. Before signing the informed consent form, participants will receive comprehensive information about the trial. To ensure maximum confidentiality, data will be processed anonymously, with only participant codes stored in the central database. Personal information of potential and registered participants beyond the necessary scope will not be collected, shared, or retained. The study results will be published in peer-reviewed journals.

## Discussion

PD is prevalent among women of reproductive age, yet often fail to receive timely and adequate treatment. This not only adversely impacts their quality of life but may also give rise to various psychological health issues. Therefore, the prompt alleviation of PD is a primary concern for female patients. The results of this study are expected to offer a simple and rapid treatment for people with acute PD.

The production of prostaglandins by the cyclooxygenase pathway is an important cause of PD, especially as a result of increased prostaglandins (PGs). Increased PGs cause uterine contractions that restrict blood flow and lead to the production of anaerobic metabolites that stimulate pain receptors (8). NSAIDs can inhibit the synthesis of PGs to achieve analgesic effects (33). Considering the short time effectiveness of medication and the accompanying side effects, more and more women are preferring acupuncture. The study has shown that acupuncture can significantly increase *β*-endorphin levels and produce endogenous analgesic effects to relieve PD (34). Acupuncture can also help to inhibit uterine contractions and lower PGs thereby reducing cramps and other symptoms of menstrual cramps (35, 36). In some studies, it was mentioned that the treatment time of acupuncture in acute pain relief was about 30 min, and the observation time was set to 10 min to obtain optimal efficacy in a shorter period and to better verify the immediate analgesic effect of acupuncture.

According to the theory of Chinese medicine meridians, the three meridians of liver, spleen, and kidney are closely related to the PD, and the acupoints prescription for this study are mainly selected from the meridians of these three organs, including Sanyinjiao (SP6), Diji (SP8), Gongsun (SP4), Jiexi (ST4), Zusanli (ST36), Taichong (LR3) (19, 37, 38).

For this study, a commonly utilized placebo technique involving the use of blunt non-penetrating needle stimulation is chosen as the control intervention (39, 40). To mitigate potential bias arising from unblinding, acupuncturists are instructed to maintain minimal contact between the blunt needle and the acupoints, thereby inducing only a slight sensation of pricking to minimize variations in needle perception. Furthermore, a blinded assessment scale will be developed to evaluate the efficacy of the blinding methodology. For the study time, the general drug onset time is 15 min (9), while some studies have shown that the onset time of acupuncture and SA is 10 min (41); to better validate the immediate analgesic efficacy of acupuncture, the study is set up for 10 min. And the treatment duration is short, at the end of the treatment, when acupuncture could not provide enough pain relief, we could use traditional medicines in time. The use of emergency analgesics is counted in both groups and the remedial analgesia rate is calculated.

The VAS is a commonly used scale for the clinical evaluation of pain and provides a simple, intuitive, continuous, and sensitive way to quantify the subjective perception of pain for PD assessment. The objective of this randomized controlled study is to investigate the immediate analgesic effect of acupuncture within 10 min in treating acute PD, by comparing the outcomes between the Acupuncture group and the SA group. The findings will not only clarify the efficacy of acupuncture as a fast-acting, low-risk, non-pharmacologic intervention but also provide evidence-based support for its integration into clinical practice. Specifically, the results may inform clinical decision-making and promote the use of acupuncture as a front-line treatment strategy in emergency or first-contact care settings, such as gynecological outpatient clinics, urgent care units, or primary health centers, particularly for patients for whom pharmacological treatments are unsuitable or who have concerns about them.

Despite efforts to improve the validity of this study, there are some limitations. Firstly, the lack of a positive control group prevents direct comparisons between acupuncture and other therapies. Second, the single-centre design may limit the generalisability of the results, and multi-centre validation is needed. Third, in this study, sham needles are blunt-tipped and touch the skin without penetration, a commonly used control method. Nevertheless, skin contact may still induce physiological responses, so residual non-specific effects cannot be fully excluded. This is a potential limitation of our sham needle design and should be considered when interpreting the study results. Finally, Although the focus of this study is to assess the immediate analgesic effects of acupuncture on patients with acute primary dysmenorrhea, the lack of short-term or delayed follow-up observations limited the interpretation of the intervention’s sustained and cumulative analgesic effects. Future studies will conduct follow-ups over a longer observation period to explore whether the analgesic effects of acupuncture can persist beyond immediate treatment and further clarify its potential cumulative effects.

In short, PD patients are in urgent need of rapid pain relief. Although acupuncture is considered an effective treatment, there is a lack of research evidence to support it. Therefore, the results of this study will provide an easy and rapid treatment option for PD patients. Importantly, the results may help reduce reliance on NSAIDs by providing a safe and immediately effective non-pharmacological alternative.
