# Supplementary material for: Immediate analgesic effect of acupuncture for acute primary dysmenorrhea: protocol of a randomized controlled trial
Source: Front Med (Lausanne). 2025 Sep 10;12:1644666. doi: 10.3389/fmed.2025.1644666 (PMC12457383; doi:10.3389/fmed.2025.1644666)
Supplement: Supplementary file 1 [file Supplementary_file_1.docx]

**Informed Consent**

**Informed Page**

**Name of Project:** Clinical study of the immediate analgesic effect of acupuncture in 10 minutes for acute primary dysmenorrhea.

**Source of project:** This study is supported by the “Liang Fanrong Expert Workstation” of Yunnan Province-Yunnan Science and Technology Programme (202305AF150072), the Yunnan Ten Thousand Talents Plan Youth Project (YNWR-QNBJ-2019-257), the “Liu Zili Famous Doctor” special talent program of the Yunnan Provincial Xing Dian Talent Support Program (Yunnan Party Talent Office 2022 No. 18), and the Yunnan Province High-level Chinese Medicine Talents - Chinese Medicine Acupuncture Discipline Leader Cultivation Objects Projec (Yunnan Provincial Health Commission 2024 No.7).

**Project research organization:** School of Second Clinical medicine/The Second Affiliated Hospital, Yunnan University of Chinese Medicine, Kunming, China.

**Research leader**: Taipin Guo

Dear patients,

First of all, thank you for your interest in our clinical research! We would like to invite you to participate in a clinical study on the clinical study of the immediate analgesic effect of acupuncture in 10 minutes for acute primary dysmenorrhea. This study has been approved by the Medical Ethics Committee of the Second Affiliated Hospital of Yunnan University of Chinese Medicine. Before you decide whether or not to participate in this study, please read the following as carefully as possible. It will help you understand the study and why it is being conducted, the procedures and duration of the study, and the benefits, risks, and discomforts that may be brought to you by participating in the study. If you wish, you can also discuss it with your relatives and friends or ask your doctor to give explanations to help you make your decision.

**Research introduction**

1. **Research background and research purposes**
2. **Research background**

Primary Dysmenorrhea (PD) is a functional pain disorder without organic pathology, characterized by severe cramping lower abdominal pain during or before menstruation. It has been a common gynecological condition that troubles women, and globally, the prevalence among women of reproductive age ranges from 16% to 94%. Severe cases may be accompanied by additional symptoms such as nausea, vomiting, diarrhea, weakness, and even fainting. Moderate to severe acute PD can be debilitating for women of childbearing age, reducing their quality of life, affecting their productivity at work and school, and potentially causing additional psychological burdens. In Europe, PD results in the loss of 3.6 million quality-adjusted life years annually, which is comparable to the impact of chronic conditions such as asthma or chronic migraines.

Considering the short time effectiveness of medication and the accompanying side effects, more and more women are preferring acupuncture. The study has shown that acupuncture can significantly increase β-endorphin levels and produce endogenous analgesic effects to relieve PD. Acupuncture can also help to inhibit uterine contractions and lower PGs thereby reducing cramps and other symptoms of menstrual cramps.

**2. Research purposes**

This study aimed to assess the immediate analgesic effect of acupuncture in 10 minutes for acute primary dysmenorrhea.

1. **Study expected number of participants**

This study is expected to include 80 patients with acute PD.

**II. Who can participate in this study?**

(1) Meeting the diagnostic criteria for PD and experiencing an acute episode;

(2) Age 14 to 35 years old;

(3) 4 ≤ pain visual analogue scale (VAS) score ≤9;

(4) Have not received acupuncture treatment or taken analgesic drugs after this episode;

(5) Voluntary participation in this study and signed informed consent, with informed family consent required for those under 18 years of age;

(6) No other studies have participated.

**III. Who is not suitable for research?**

(1) Participants with combined severe life-threatening primary diseases and other acute abdominal pain;

(2) Participants with severe anxiety, depression, insomnia, and other psychiatric disorders;

(3) Participants who are afraid of acupuncture, do not cooperate with treatment or are prone to acupuncture dizziness.

**IV. What will be done if you participate in the research?**

If you meet the inclusion criteria and agree to participate, you will first undergo relevant tests to ensure that you meet all the requirements for study participation.

**1. Before you are included in the study, you will undergo the following tests to determine if you can participate in the study.**

Your medical history, clinical signs, and symptoms will be interviewed and recorded; you will also be instructed to complete a visual analogue scale score (VAS) to determine whether you meet the inclusion criteria.

**2. If you meet the inclusion criteria through the above screening, the study will be conducted according to the following steps:**

(1) The trial will be divided into 2 groups. At the beginning of the study, your doctor will decide which group you will receive based on the random numbers generated by a computer.

(2) Acupuncture needle: use the Huatuo brand disposable acupuncture needles produced by Suzhou Medical Supplies Factory Ltd, China. Manufacturer's license number: Su Food and Drug Administration of Machinery Production 20010020; Registration certificate number: 201622770970. specifications of acupuncture are 0.30 x (25mm, 40mm).

**3. Other matters requiring your cooperation**

Throughout the treatment process, it's essential to collaborate with your doctor by completing relevant scales, providing truthful answers to their questions, cooperating with their instructions, and offering feedback on your condition.

**V. Possible benefits of participating in the study**

You may derive benefits from participating in this study, such as improving your condition and receiving valuable health education on the prevention and treatment of PD.

**VI. Adverse reactions, risks, and protective measures for participating in the study**

You may have soreness, numbness, heaviness, and swelling during the acupuncture process, which are all normal reactions to acupuncture. There may be adverse reactions after needling, but they are rare and mild. You may feel dizzy during needling due to your physical condition or emotional stress, which can be relieved after stopping needling and taking proper rest; bleeding and haematoma may occur after needling, which will disappear after local pressure; however, if infection occurs at the site of needling, your doctor will deal with it promptly.

If you experience any discomfort, new changes in your condition, or any unforeseen circumstances during the study period, whether or not they are related to the acupuncture treatment, you should inform your doctor promptly and he/she will make a judgement and give appropriate medical treatment.

**VII. Treatment options available to you other than participating in this study**

Your doctor will discuss with you the other treatment options currently available for your condition, including the corresponding risks and benefits. For primary dysmenorrhea, there are currently anti-inflammatory and analgesic drugs, mainly non-steroidal anti-inflammatory drugs (NSAIDs), which are effective but have side effects such as gastrointestinal bleeding, stomach ulcers and cerebrovascular accidents.

**VIII. The relevant costs**

All the costs of this project are supported by the “Liang Fanrong Expert Workstation” of Yunnan Province-Yunnan Science and Technology Programme (202305AF150072), the Yunnan Ten Thousand Talents Plan Youth Project (YNWR-QNBJ-2019-257), the “Liu Zili Famous Doctor” special talent program of the Yunnan Provincial Xing Dian Talent Support Program (Yunnan Party Talent Office 2022 No. 18), and the Yunnan Province High-level Chinese Medicine Talents - Chinese Medicine Acupuncture Discipline Leader Cultivation Objects Projects (Yunnan Provincial Health Commission 2024 No.7). If you participate in this study, you will receive free acupuncture treatment during the study period. This study will only observe the efficacy of the treatment once, and if the subsequent relief is not obvious, you can have 2 free acupuncture treatments. Doctors will make every effort to prevent and treat any harm that may occur as a result of this study. If an adverse event occurs during the clinical trial, a committee of medical experts will determine whether it is related to the acupuncture treatment or the study process. The sponsor will provide the cost of treatment and financial compensation for any harm related to the trial process in accordance with the provisions of China's Code of Practice for the Quality Management of Pharmaceutical Clinical Trials.

During the treatment period, if you have a combination of other medical conditions, the treatment and examination will not be free of charge.

**IX.The confidentiality of clinical data**

Your medical records (study charts/CRFs, etc.) will be kept intact at the hospital where you are seen. The investigator, ethics committee and drug regulatory authorities will be given access to your medical records. Any public reporting of the results of this study will not disclose your identity. We will make every effort to protect the privacy of your personal medical information to the extent permitted by law.

**X. You can voluntarily choose to participate in research and withdraw from the study**

Whether or not to participate in the research is entirely up to you. You may decline to participate in the study or withdraw from the study at any time during the study, which will not affect your relationship with the doctor and will not affect your medical or other benefits.

Your physician will promptly notify you if an important subject-related event or information occurs during the study that may affect your willingness to continue participating in the study.

**XI. What should I do now?**

Participation in this clinical study is based on a completely voluntary principle and needs to be carried out with your consent and signed informed consent. Whether or not you participate in this clinical study depends entirely on your wishes. You have the right to suspend and withdraw from this research treatment at any time. Exiting this study will not affect your medical treatment.

Your physician may suspend your participation in this study in advance if: Your health condition is not suitable for continued participation, or you may not comply with the research program requirements.

The doctor will promptly notify you or your legal representative if there is medical information that may affect your willingness to continue your research during the study. Before you decide to participate in this study, please ask your life as much as possible until you fully understand this test study.

If you have any questions, suggestions, or complaints about this study, please do not hesitate to discuss them with the research team, whose contact details can be found on the signature page. If you feel inconvenienced to communicate with the research team, you can consult or complain to the Medical Ethics Committee of the Second Affiliated Hospital of Yunnan University of Traditional Chinese Medicine. Ethics Committee contact number:15125208547.

Thank you for reading the above material. If you decide to take part in this study, please let your doctor know and he/she will make all the arrangements for you to study.

**Informed Consent**

**Signature Page**

1. I have carefully read the contents of the informed consent form, and the researchers have answered my questions.

2. Having fully understood the purpose, methods, possible therapeutic benefits and risks to be encountered and other terms of this clinical study as mentioned in the informed consent form, I voluntarily participate in this study and promise to cooperate fully with the investigators.

3. I understand that I can withdraw from the study at any time and I do not need any reason. The medical services I receive and the legal rights I enjoy are not affected at all.

Finally, I decided to agree to participate in this study and to ensure compliance with my doctor’s advice.

Subject Signature: _________________ Date: _________________

Contact Number: _________________

I have explained fully detail to the subjects, including the potential risks.

Doctor/Researcher Signature: _________________ Date: _________________

Contact Number: _________________

**知情同意书**

**告知页**

**项目名称：**针刺10分钟即刻镇痛对急性原发性痛经的疗效的临床研究

**项目来源：**云南省科技计划项目-云南省梁繁荣专家工作站（202305AF150072）、云南省万人计划青年拔尖人才项目（YNWR-QNBJ-2019-257）、云南省兴滇英才支持计划“刘自力名医”专项（云党人才办[2022]18号）、云南省高层次中医药人才-中医针灸学科带头人培养对象项目(云南省卫生健康委2024 No.7)。

**课题研究单位**：云南中医药大学针灸推拿康复学院/第二附属医院

**项目负责人**：郭太品

**亲爱的患者:**

首先，感谢您对我们这项临床研究的关注！我们将邀请您参加一项“针刺10分钟即刻镇痛对急性原发性痛经的疗效”的临床研究。本研究已通过云南中医药大学第二附属医院医学伦理委员会审核，同意进行临床研究。在您决定是否参加这项研究之前，请尽可能仔细阅读以下内容。它可以帮助您了解该项研究以及为何要进行这项研究，研究的程序和期限，参加研究后可能给您带来的益处、风险和不适。如果您愿意，您也可以和您的亲属、朋友一起讨论，或者请医生给予解释，帮助您做出决定。

**研究介绍**

**一、研究背景和目的**

**1. 研究背景**

原发性痛经(PD)是一种无器质性病理的功能性疼痛障碍，其特征是在月经期间或月经前出现严重的痉挛性下腹痛。它一直是困扰女性的一种常见妇科疾病，在全球范围内，育龄妇女的患病率从16%到94%不等。严重者可能伴有其他症状，如恶心、呕吐、腹泻、虚弱甚至昏厥。中度至重度急性PD可使育龄妇女衰弱，降低她们的生活质量，影响她们的工作和学习效率，并可能造成额外的心理负担。在欧洲，PD每年导致360万质量调整生命年的损失，这与哮喘或慢性偏头痛等慢性疾病的影响相当。

考虑到药物见效短，副作用大，越来越多的女性选择针灸。研究表明，针刺可显著提高β-内啡肽水平，产生内源性镇痛作用，缓解PD。针灸还可以帮助抑制子宫收缩和降低PGs，从而减少痛经和其他痛经症状。

1. **研究目的**

本研究旨在评估针刺10分钟内对急性原发性痛经的即时镇痛效果。

1. **研究预计纳入参试者例数**

本研究预计纳入80例急性痛经患者。

**二、哪些人能参加这项研究？**

1. 符合原发性痛经诊断标准，且正处于痛经急性发作期；
2. 年龄14~35岁；
3. 疼痛视觉模拟评分4 ≤（VAS）≤9分；
4. 未接受过针刺治疗，未服用过镇痛药物；
5. 自愿参加本研究，并签署知情同意书，18岁以下需取得家属知情同意；
6. 没有参与其他研究。

**三、哪些人不宜参加本研究**

1. 合并严重危及生命的原发性疾病以及其他急性腹痛患者；
2. 伴有严重焦虑、抑郁、失眠等精神类疾病者；

（3）畏惧针灸，不配合治疗，或容易晕针者；

**四、如果参加研究将要做什么?**

1. **在您入选研究前：**

将对您的病史、临床体征和症状进行面谈和记录;您还将被要求完成视觉模拟评分(VAS)，以确定您是否符合纳入标准。

1. **若您通过以上筛查符合纳入标准，将按以下步骤进行研究**

（1）试验将分为2组。在研究开始时，你的医生将根据电脑提供的随机数决定你将接受哪一组。

（2）针灸针:使用中国苏州医疗用品厂有限公司生产的华佗牌一次性针灸针。生产企业许可证号:苏食品药品监督管理局机械生产20010020;注册证号码:201622770970。针灸规格为0.30 × (25mm、40mm)。

1. **需要您配合的其他事项**

在治疗过程中，您需要配合医生完成相关量表填写，如实回答医生提问的问题，配合医生，对您病情进行反馈。

**五、参加研究可能的受益**

您可能会在本项研究中获益。这些好处包括您的病情有望改善，以及接受关于预防急性痛经的健康教育。

**六、参加研究可能的不良反应、风险和不适、不方便**

针刺过程中您可能会有酸、麻、重、胀的感觉，这均为针刺的正常反应。针刺后可能存在不良反应，但较少而轻微，针刺时可能因为您的体质问题或情绪紧张出现晕针现象，停止针刺和适当休息后可缓解；针刺后可能出现出血、血肿等现象，经局部按压后可消失；但如果针刺部位出现感染，您的医生会及时处理。

如果在研究期间您出现任何不适，或病情发生新的变化，或任何意外情况，不管是否与针刺治疗有关，均应及时通知您的医生，他/她将对此作出判断并给与适当的医疗处理。

**七、除参加本研究外，您可选的其他治疗**

您的医生将与您讨论目前针对您的病情可选择的其他治疗方案，包括相应的风险和益处。针对急性腰扭伤的患者，目前可以选择非甾体类抗炎药（NSAIDS）为主的消炎镇痛药进行治疗，作用效果较好，主要存在胃肠道出血、胃溃疡、脑血管意外等副作用。

**八、有关费用**

本课题所有费用由云南省科技计划项目-云南省梁繁荣专家工作站（202305AF150072）、云南省万人计划青年拔尖人才项目（YNWR-QNBJ-2019-257）、云南省兴滇英才支持计划“刘自力名医”专项（云党人才办[2022]18号）、云南省高层次中医药人才-中医针灸学科带头人培养对象项目(云南省卫生健康委2024 No.7)资助。如您参见本研究，在研究期间，将得到相关免费针刺治疗。本研究仅观察治疗一次的疗效，如疗效缓解不明显，可免费做2次针刺治疗。医生将尽全力预防和治疗由于本研究可能带来的伤害。如果在临床试验中出现不良事件，医学专家委员会将会鉴定其是否与针刺治疗或研究过程有关。申办者将按照我国《药物临床试验质量管理规范》的规定对与试验过程中相关的损害提供治疗的费用及相应的经济补偿。

在治疗期间，如果您同时合并其他疾病所需的治疗和检查，将不在免费的范围之内。

**九、个人信息是保密的吗？**

您的医疗记录（研究病历/CRF等）将完整地保存在您所就诊的医院。研究者、伦理委员会和药品监督管理部门将被允许查阅您的医疗记录。任何有关本项研究结果的公开报告将不会披露您的个人身份。我们将在法律允许的范围内，尽一切努力保护您个人医疗资料的隐私。

**十、可以自愿选择参加研究和中途退出研究**

是否参加研究完全取决于您的意愿。您可以拒绝参加此项研究，或在研究过程中的任何时间退出本研究，这都不会影响您和医生间的关系，都不会影响您的医疗待遇与权益，或有其他方面利益的损失。

研究过程中如果发生与受试者相关的重要事件或信息，可能会影响您继续参加研究的意愿时，您的医生将及时通知您。

1. **怎样获得更多的信息?**

参加本项临床研究，本着完全自愿的原则，需要在您同意并签署知情同意书的前提下进行。是否参加本项临床研究，完全取决于您本人的意愿，您有权在任何时候选择中止和退出本项研究性治疗，退出本研究并不会影响您的医疗待遇。

您的医师可以在下列情况下提前中止您继续参加本项研究：您的健康状况不适合继续参加，或者您不能遵守研究方案的要求。

如在研究过程中出现可能影响您继续参加研究意愿的医学信息，医生将及时通知您或者您的法定代表。在您做出参加本研究的决定前，请尽可能向您的医生询问有关问题，直至您对本项试验研究完全理解。

如您对这项研究存在任何疑问、建议或投诉，请及时与研究团队讨论，联系方式见签字页。如您感觉不便与研究团队沟通，可向云南中医药大学第二附属医院医学伦理委员会进行咨询或投诉。伦理委员会联系电话:15125208547。

感谢您阅读以上材料。如果您决定参加本项研究，请告诉您的医生，他/她会为您安排切有关研究的事务。

**知情同意书**

**签字页**

1. 我已经仔细阅读了知情同意书告知页的内容，研究者已解答了我提出的疑问。

2. 我在充分理解了知情同意书提及的本项临床研究的目的、方法、可能获得的治疗利益和可能遇到的风险以及其他条款后，自愿参加此项研究，并承诺与研究者充分合作。

3. 我明白我可在任何时候退出研究，并且不需要任何理由，我得到的医疗服务和享有的法律权利不受任何影响。

最后，我决定同意参加本项研究，并保证遵从医嘱。

受试者签名:_________________ 日期：_______年_______月_______

联系电话:___________________

我确认已向患者解释了本研究的详细情况，包括其权力及可能的受益和风险。

医生/研究者签名:____________ 日期：_______年_______月________

联系电话:___________________
